# Cases of Colloid Tumor of the Abdomen

**Published:** 1860-09

**Authors:** S. D. Gross

**Affiliations:** Professor of Surgery in the Jefferson Medical College of Philadelphia


### ART. V.—*Cases of Colloid Tumor of the Abdomen.* By S. D. GROSS, M.D., Professor of Surgery in the Jefferson Medical College of Philadelphia.

A GENTLEMAN, aged forty-five, married, of temperate and industrious habits, and a resident for many years of Louisville, had been the subject of occasional colicky pains, with a sense of fullness in the abdomen for upwards of four months, when, early in November, 1854, he applied to me for advice. On inquiry, I was told that he had been obliged for some time past to unbutton his pantaloons occasionally, in order to render himself comfortable, the constriction of the waistband causing a sense of tightness in the abdomen and some difficulty in breathing. His general health was as good as usual; he had a fair amount of appetite, his strength was unimpaired, and he was able to attend to his business. On examining the abdomen, I found it to be exceedingly hard and prominent, owing to the presence of a large tumor, extending, on the one hand, from the epigastric region low down into the pelvic, and, on the other, on each side, deeply into the loin. Inferiorly it was somewhat of a pyramidal shape, with sharp, well defined edges, under which it was easy to insinuate the fingers. The umbilical region was very prominent, and the whole abdomen was excessively hard. The tumor was free from pain, and immovable. What was very remarkable, the patient was entirely unconscious of its existence. Having seen a similar case a number of years previously, I became at once satisfied that it was of a colloid character.

The gentleman, warned of his danger, abandoned his business, and began the use of a gentle course of alteratives, with mild laxatives, strict attention to the diet, and a sorbefacient ointment to the abdomen. In pleasant weather he was permitted to go out into the open air, generally on foot, as riding in his carriage was productive of soreness in the abdomen. My former colleague, Professor Austin Flint, saw him with me in February. The only perceptible change during the last three months was the existence of a small quantity of water behind the umbilicus, the collection being about the diameter of a breakfast plate, with distinct fluctuation, and an occasional twinge of pain. In the spring, as the weather became warm, he walked out more frequently, and early in May went to Crab Orchard Springs, Kentucky, where he spent a month, without, however, any apparent benefit. In July he visited several of the principal watering-places in Virginia. While at the Hot Springs, early in September, he was suddenly taken alarmingly ill with pain in the abdomen, attended with excessive tenderness on pressure, high fever, and disorder of the digestive organs, as nausea, diarrhœa, and want of appetite. After suffering in this way for about two months, he finally reached home on the fourth of November, in a state of the most perfect emaciation and exhaustion. The tumor had apparently neither increased nor diminished in size, shape, or consistence, but it was somewhat more tender on pressure, and its surface was a little more lobulated. No fluctuation could be discovered anywhere. The patient lingered until the morning of the eighth, when he expired.

The body was examined nine hours after death, in presence of Professor Flint.

The peritoneal cavity was completely obliterated, except in a small space corresponding with the upper part of the right lobe of the liver. The walls of the abdomen adhered everywhere to the surface of the tumor, and in attempting to separate them the knife repeatedly entered the folds of the small bowel. The tumor occupied the whole abdominal cavity, from the neck of the bladder to the diaphragm, which was pushed high up toward the chest. All the viscera were involved in it, though free from colloid. The folds of the bowels, large and small, were included in and compressed by it; the stomach was in the same condition, and was apparently much reduced in size; its mucous membrane was slightly red and thickened; the pylorus was sound. The liver was natural, but the greater portion of it was involved in the tumor; the inferior and right portions alone were free. The gall-bladder had also suffered from compression, and contained about an ounce of dark bile. The spleen was completely imprisoned, as was also the pancreas; both organs being sound. The kidneys lay exterior to the tumor, and were perfectly natural. The bladder was contracted, and compressed at its neck, where there was a large mass of colloid. The prostate gland was normal.

The vessels of the abdomen, the lungs, and heart were healthy. The brain was not examined.

One of the most remarkable features of this immense tumor was the existence of a large pouch in front of the bowels, more than nine inches in diameter, and capable of holding more than a quart of fluid. Its surface had a rough, muddy appearance, as if it had contained fæcal matter, and been covered with coagulating lymph. How it had been formed is a subject of conjecture. The most plausible supposition is that it had been induced by the escape of fæces, probably a very small quantity, the result of ulceration and perforation of the bowel, leading to inflammation, and the deposition of plastic matter. No such opening, however, was detected. Was the fluid present during the preceding winter and spring contained in this pouch? or was the pouch formed in September, during the severe illness, soon after the arrival of the patient at the Hot Springs?

The colloid character of the morbid growth was well marked throughout. The cysts or cavities varied from the size of a small currant to that of a hazel-nut, and were for the most part of a spherical shape. Their contents were of a thick, jelly-like consistence, and of a white appearance, with a shade of greenish.

The tumor was very firm, and grated distinctly under the knife. Its thickness in several places was upwards of three inches, while its breadth and length were each upwards of one foot. As far as could be determined during the dissection, it seemed to have been developed in the omentum, all trace of which was effaced.

Another enormous colloid tumor came under my observation in 1840, in a laboring man, aged forty-nine, whose body I examined along with the late Dr. Mason, of Cincinnati. The following abstract of the case is copied from my "Elements of Pathological Anatomy," p. 190, third edition, 1857:—

"It extended from the pelvis to the liver and diaphragm, surrounded the colon and part of the stomach, and concealed from view nearly the whole of the abdominal viscera. Its thickness was from two and a half to three inches; in length it measured nearly one foot, and in breadth more than eight inches. Its weight was estimated at twenty-five pounds. It was composed of thousands of agglomerated masses, from the size of a grain of mustard to that of a hickory-nut, and seemed to have been developed in the peritoneum, or in the great omentum, the latter of which was completely effaced. The liver was smaller than natural, probably from the pressure exerted upon it by the tumor, but in other respects it was perfectly sound, as were likewise the other viscera, both abdominal and thoracic. The disease was first noticed in July, 1837, as a small, hard, circumscribed tumor, just above the pubes."

In 1858, I exhibited at a meeting of the Philadelphia Pathological Society a specimen of colloid growth, presented to me by Dr. Van Wyck, of this city, being a portion of an enormous tumor occupying the entire peritoneal cavity. It weighed upwards of twenty pounds. The patient was a man of fifty-four years of age, a tailor by trade, of spare make, and temperate habits.

"The morbid growth had been first observed about two years before death, having commenced in the left iliac region, from which it gradually extended in different directions until it filled the whole abdomen. Dr. Van Wyck had attended the man for about twelve months, when the tumor was nearly as large as at the post-mortem examination. The principal symptom was an extreme sense of weight and oppression in the abdomen, attended with considerable dyspnœa, especially when he suffered from constipation, to which he was very subject. He was but little emaciated, and there was hardly any œdema of the extremities. On dissection, it was found that the whole peritoneal cavity was occupied by an enormous colloid mass, reaching from the pelvis up to the diaphragm, which it had pushed high up into the chest, covering completely the abdominal viscera, and effacing all trace of the serous cavity. It weighed upwards of twenty pounds, and was fully two inches and a half in thickness, whitish in its appearance, and composed of numerous characteristic cysts, from the volume of a mustard-seed to that of a small hickory-nut. All the abdominal, pelvic, and thoracic viscera were sound. A microscopic examination by Dr. Da Costa showed small cells, indistinctly nucleated, fibres, and an amorphous basement substance."

